# Laser interstitial thermal therapy using the Leksell Stereotactic System and a diagnostic MRI suite: how I do it

**DOI:** 10.1007/s00701-022-05461-x

**Published:** 2022-12-31

**Authors:** Margret Jensdottir, Ulrika Sandvik, Michael Fagerlund, Jiri Bartek

**Affiliations:** 1 Department of Clinical Neuroscience, Section for Neurosurgery, Karolinska Institute and Department of Neurosurgery, Karolinska University Hospital, Stockholm, Sweden; 2 Department of Neuroradiology, Karolinska University Hospital, Stockholm, Sweden; 3 Department of Neurosurgery, Rigshospitalet, Copenhagen, Denmark

**Keywords:** LITT, Laser interstitial thermal therapy, Neurosurgery, Stereotactic laser ablation, Stereotactic frame, Minimally invasive neurosurgery

## Abstract

**Background:**

Laser interstitial thermal therapy (LITT) is a stereotactic neurosurgical procedure used to treat neoplastic and epileptogenic lesions in the brain. A variety of advanced technological instruments such as frameless navigation systems, robotics, and intraoperative MRI are often described in this context, although the surgical procedure can also be performed using a standard stereotactic setup and a diagnostic MRI suite.

**Methods:**

We report on our experience and a surgical technique using a Leksell stereotactic frame and a diagnostic MRI suite to perform LITT.

**Conclusion:**

LITT can be safely performed using the Leksell frame and a diagnostic MRI suite, making the technique available even to neuro-oncology centers without advanced technological setup.

**Supplementary information:**

The online version contains supplementary material available at 10.1007/s00701-022-05461-x.

## Relevant surgical anatomy

Laser interstitial thermal therapy (LITT) is a minimally invasive treatment modality for ablation of neoplastic or epileptic lesions by thermal damage. The introduction of reproducible MRI thermometry, with real-time monitoring of thermal damage, allows for a focused ablation while lowering the risk of injury to surrounding structures [2, 4, 6]. LITT offers the advantage to treat an otherwise difficult to reach central nervous system lesion in or near eloquent areas. The potential treatment diameter is around 2–3 cm. With critically located lesions, the precision of laser catheter placement is of utmost importance, to ensure adequate coverage while avoiding collateral damage [6, 8].

## Description of the technique

Several stereotactical systems are available for laser catheter placement and the LITT technique is usually associated with an advanced technical setup such as an intraoperative MRI, frameless stereotactic systems, and robotics [2, 8, 3]. Nevertheless, LITT can also be performed using more widely available equipment, such as a stereotactic frame and a diagnostic MRI suite.

The Visualase™ (Medtronic, Inc., Minneapolis MN) LITT device consists of a laser diode (980 nm wavelength, 15-W), laser fiber, and applicator. The diffusing fiber optic comes in two different lengths of energy output: 3 or 10 mm. A cooling channel allows for circulating sterile saline to assure probe tip cooling. A bone anchor is affixed through a 3.2-mm twist drill hole in the skull to retain the laser applicator along the intended trajectory.

**Fig. 1:**
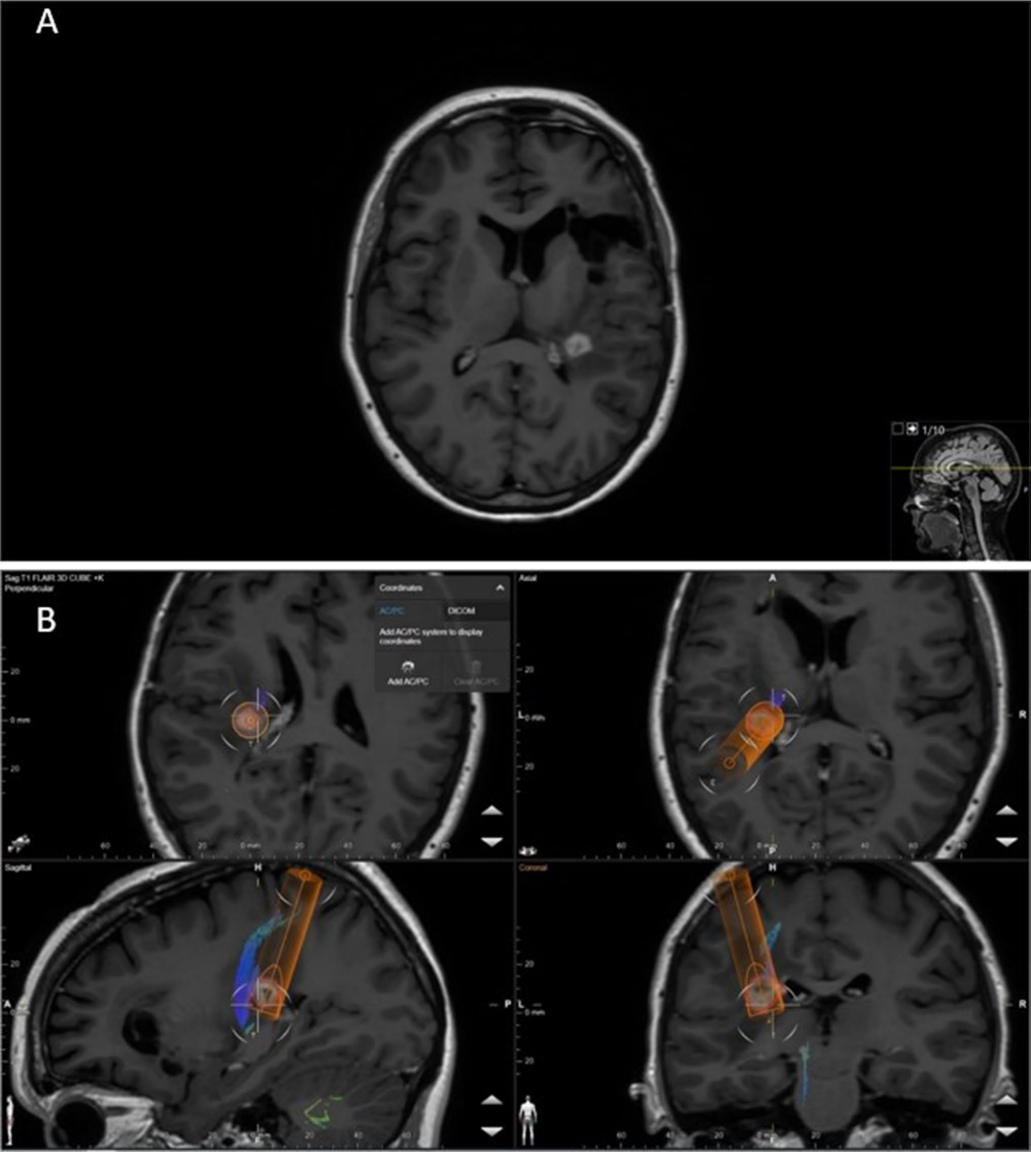
Preplanning. **A** Contrast-enhanced T1 3D volume MRI (BRAVO) sequence shows a tumor recurrence in a patient previously operated for a low-grade glioma in the left fronto-insular region with a new contrast-enhancing lesion near the basal ganglia and cortico-spinal tract (CST). **B** Preplanning with a volumetry of the lesion (red border), target (T), and entry point (E). A DTI can be incorporated for tractography of the CST (blue), when choosing a suitable entry point for a trajectory and ablation sparing the eloquent areas of the CST. A simulation of the intended ablation can be done by creating a diameter of 10–20 mm to the trajectory (orange)

**Fig. 2:**
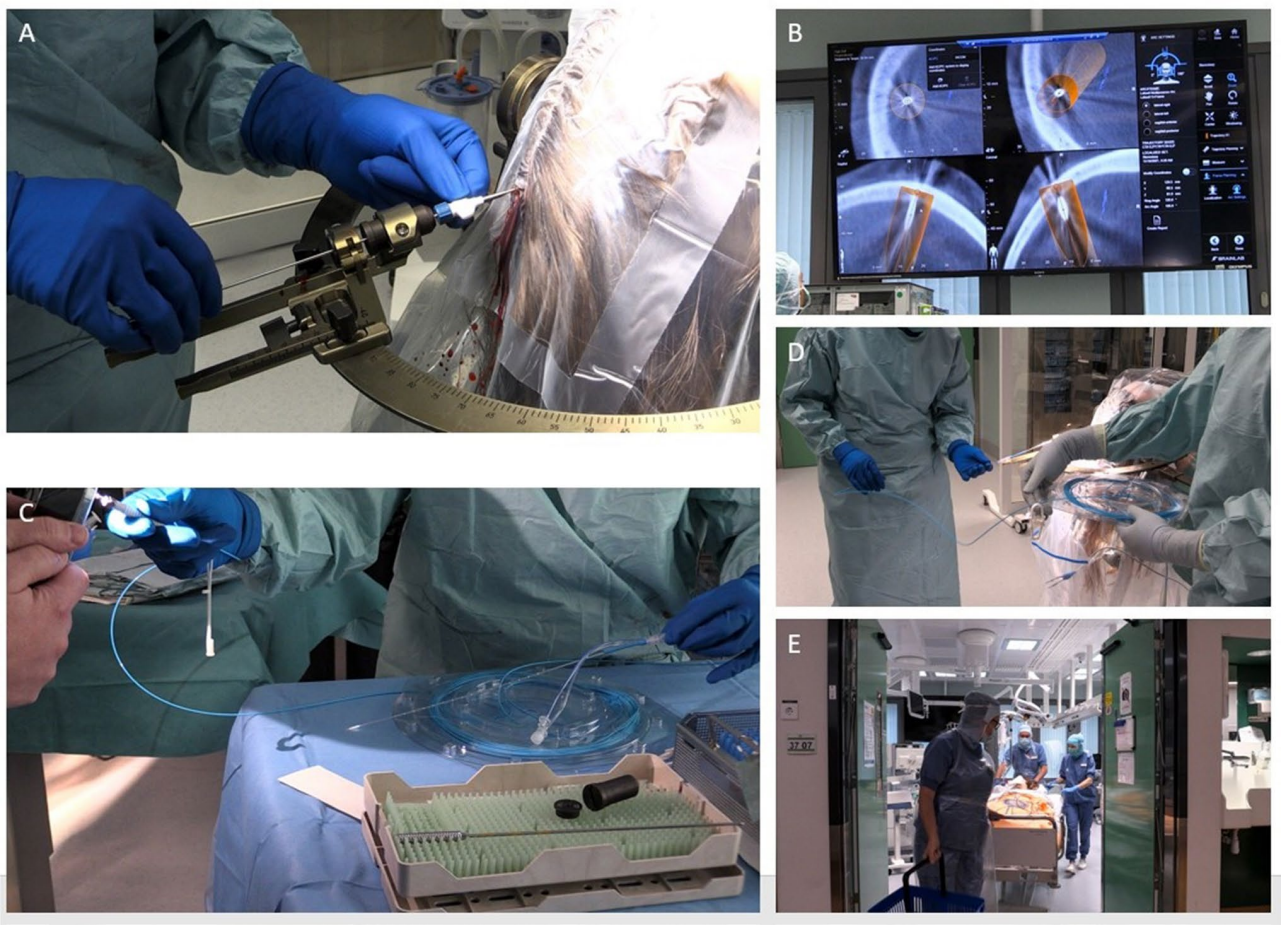
Stereotactic arc and laser catheter insertion **A**. A small scalp incision is made, followed by drilling of the bone and puncture of the dura. The entry point should have a favorable angulation to the skull, as perpendicular to the bone surface as possible. After the drilling, the rigid stylet for the bone anchor can be passed intracranially **B**. We verify the trajectory with the O arm, taking images of the rigid stylet before the bone anchor is fixed to the skull. **C**. The laser fiber is prepared and **D** the laser catheter is inserted the predetermined distance and locked in place followed by the insertion and fixation of the laser fiber. **E** The Leksell arc and frame are removed before the patient is transported to the diagnostic MRI suite

**Fig. 3:**
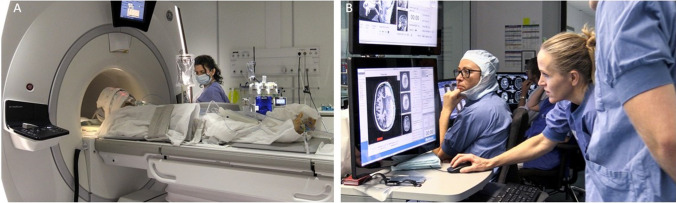
Pre-treatment MRI **A** The flex coil is positioned around the patient’s head for imaging acquisition. **B** The MRI is connected to the Visualase console. Two Tmap imaging planes, in this case axial and sagittal, are selected for continuous thermometry imaging during treatment

**Fig. 4:**
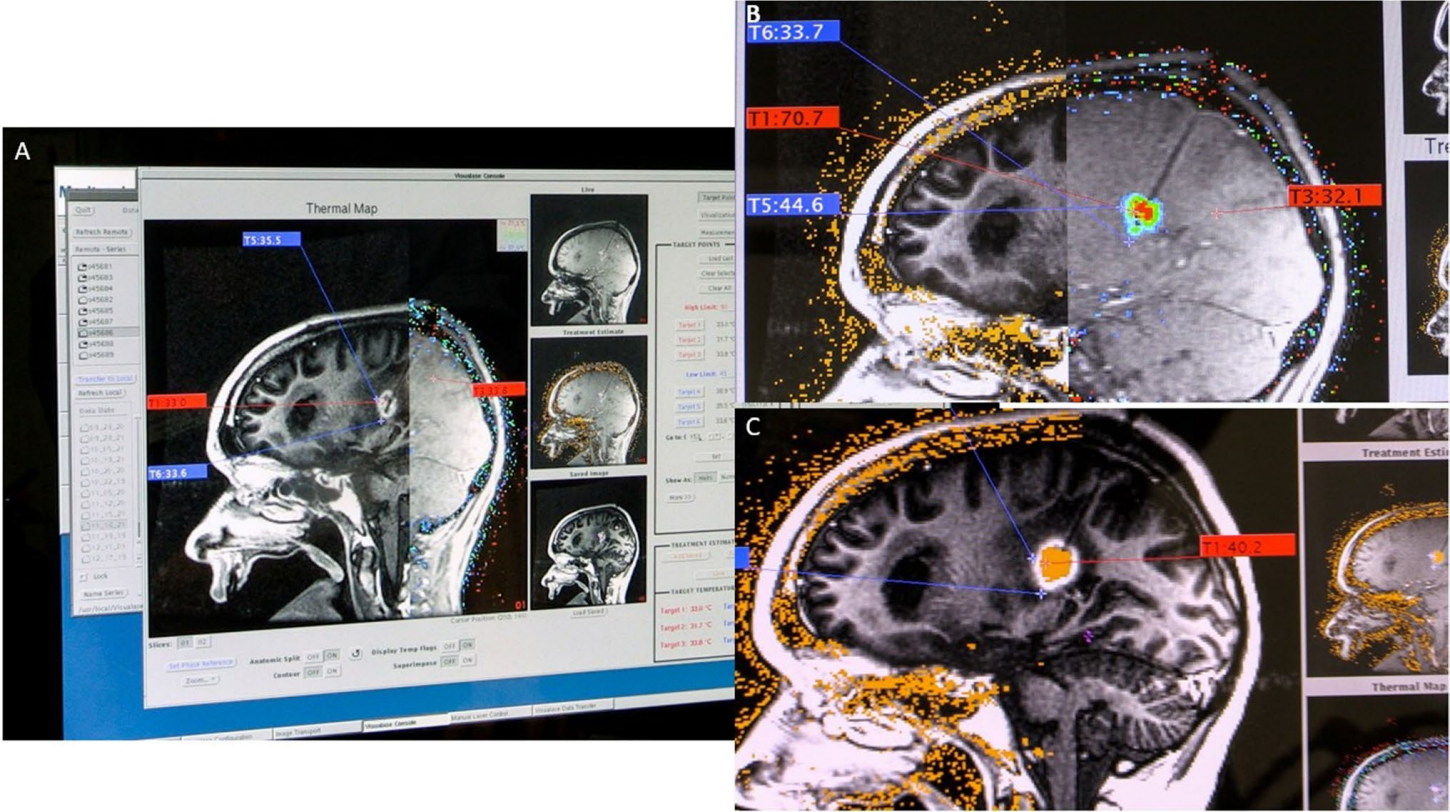
LITT ablation procedure **A**. High-temperature markers (< 90°) are set in the vicinity of the catheter tip to protect it from overheating and low-temperature markers (< 45–50°) close to critical areas to be protected (in this case the motor fiber tracts). Irrigation is started to ensure cooling of the catheter during treatment. **B**. A low-energy test dose is delivered to confirm heat distribution in the intended area. The energy is increased for the ablation itself and the tissue temperature changes are followed on the console as color changes on the imaging, while the lesional damage estimate is calculated by the system and monitored continuously on the console. **C** Contrast-enhanced T1 3D volume MRI (FSPGR) is performed after the ablation and compared to the damage map from the ablation. A contrast-enhancing peripheral ring (in addition to the contrast enhancing tumor) on the post-ablational MRI scan marks the border of the ablated volume which is seen to correspond well with the estimated damage map (orange)

The procedure can chronologically be divided into seven steps and is described accordingly in the supplementary video:

In the first step, (1) *preplanning*, the lesion is identified on a pre-treatment MRI scan. The images are exported to a neuro-navigation system, along with a preoperative CT scan. Suitable target and entry points are chosen for an optimal trajectory (Fig. 1). During the second step, (2) *stereotactic frame placement* in the OR, the stereotactic frame is fixed to the patients head under general anesthesia. Imaging for localization with an O arm (multiplane 3D imaging) is performed and coordinates for the target and entry points are retrieved. During step (3) *stereotactic arc and laser catheter insertion* (Fig. 2), the arc is positioned according to the *X*, *Y*, and *Z* coordinates for the target. A percutaneous burr hole is performed and the dura is punctured. A biopsy needle can be inserted for tissue sampling, if needed. We verify the trajectory before we affix the bone anchor and insert the laser applicator followed by the laser fiber. Finally, the Leksell arc and frame are removed and the patient is transported to the diagnostic MRI suite (Fig. 3). In the fourth step, (4) *Pre-treatment MRI,* a flex coil is placed around the patient’s head. A gadolinium contrast-enhanced MRI verifies proper positioning of the laser fiber as well as assuring that no complication, such as hemorrhage, has occurred during laser catheter insertion. The MRI is connected to the Visualase system (Fig. 4). Two imaging planes from the temperature map sequence (Tmap) are chosen for continuous thermometry during ablation. The laser fiber and cooling lines are connected to the Visualase console before the fifth step, (5) *LITT ablation procedure*, is commenced. Temperature markers are set. A low-energy test dose is delivered to confirm heat distribution in the intended area. The energy is then increased for the ablation, with simultaneous irrigation ongoing for cooling of the catheter. The tissue temperature changes as visualized by the Tmap sequence can be seen on the Visualase console, while the lesional damage estimate is calculated by the Visualase system and monitored continuously on the console (Fig. 4). The treatment can be repeated and the catheter can be adjusted along the trajectory as needed to obtain an optimal ablation. During step (6) *post treatment MRI* to evaluate the final ablation volume and to detect any adverse effects, a diagnostic contrast-enhanced MRI is performed (Fig. 4C). The final step, (7) *removal of LITT catheter* and subsequent extubation, is performed in the MRI department before transfer to the neurosurgical ward.

## Indications

LITT has been used for the treatment of malignant brain tumors and recurrent and newly diagnosed glioblastoma as well as other high-grade gliomas. The indications in neuro-oncology have continued to expand to include radioresistant brain metastasis and radionecrosis [2, 1, 5]. LITT has also become an established minimally invasive alternative to epilepsy surgery for treating different types of epileptogenic foci, such as cavernoma, mesial temporal lobe sclerosis, focal cortical dysplasia, and hypothalamic hamartoma [7, 10]. At our institution, we have treated both neoplastic and epileptogenic lesions. We find the accuracy of the stereotactic frame to be favorable in the treatment of small lesions in critical areas such as the ablation of hypothalamic hamartomas or lesions near the cortico-spinal tract as illustrated in our video.

## Limitations

Performing LITT with the Leksell frame and a diagnostic MRI suite has its advantages but also limitations. The main advantage is the broad applicability of the procedure without the need for further technical setup such as an intraoperative MRI or robotics.

An obvious limitation to performing the ablation in a diagnostic MRI suite is that it does not allow for altering of the trajectory once the Leksell frame has been removed and the patient transported to the diagnostic MRI suite. Other limitations are those inherent to the LITT treatment such as lesion size and geometry or difficult locations without a safe trajectory for catheter placement [2, 6, 8].

## How to avoid complications

Meticulous planning of the catheter placement, avoiding trajectories through critical structures and large vessels, is mandatory for a successful laser ablation and to avoid complications such as catheter malplacement and hemorrhage. Another key point to avoid laser misplacement is the angulation to the skull to avoid the drill bit from slipping on the bony curvature [9].

The risk of hemorrhagic complications during placement can further be minimized by adequate durotomy before insertion of the laser catheter.

Hyperthermic complications should be avoided by careful placement of temperature markers close to any critical structures so the laser is automatically deactivated before hyperthermia occurs in these areas.

Finally, pre-treatment imaging used for preplanning should be recent (< 1 week), especially for fast-growing tumors where a potential increase in volume might prevent coverage of the whole lesion. Similarly, attention needs to be paid to the risk of excessive edema increasing with the ablation volume [2, 5].

## Specific perioperative considerations

Preoperative workup includes MRI and CT imaging, pre-anesthesia evaluation, and coagulation tests. Postoperatively, the patient is in a recovery unit for 2–4 h before transfer to the neurosurgical ward and discharged after 24 h observation with corticosteroid tapering over the course of 2 weeks.

## Specific information for the patient

LITT offers a minimally invasive treatment, a short hospital stay, and fast recovery. However, the patient has to be informed about the possibility of adverse events, i.e., hemorrhage and neurological deficits due to misplacement of the catheter or other adverse events due to hyperthermia in critical/eloquent areas or post-procedural swelling. More importantly, the patient needs to be informed about the limitations of the treatment in terms of ablation volume. Thus, LITT is recommended for patients with lesions of appropriate size, shape, and location, assuring maximal ablation coverage while minimizing the risk of adverse events.

## Supplementary Information

Below is the link to the electronic supplementary material.

Supplementary file (MP4 92.5 mb)

## Data Availability

Data sharing not applicable to this article as no datasets were generated or analysed during the current study.
